# Construct and Compare Gene Coexpression Networks with DAPfinder and DAPview

**DOI:** 10.1186/1471-2105-12-286

**Published:** 2011-07-14

**Authors:** Jeff Skinner, Yuri Kotliarov, Sudhir Varma, Karina L Mine, Anatoly Yambartsev, Richard Simon, Yentram Huyen, Andrey Morgun

**Affiliations:** 1 Bioinformatics and Computational Biosciences Branch (BCBB), Office of Cyber Infrastructure and Computational Biology (OCICB), National Institute of Allergy and Infectious Disease (NIAID), National Institutes if Health (NIH), Bethesda, Maryland, USA; 2 Neuro-Oncology Branch (CHIAI), National Cancer Institute (NCI), National Institutes of Neurological Disorder and Stroke (NINDS), National Institutes if Health (NIH), Bethesda, Maryland, USA; 3 Setor de Imunogenética, Departamento de Pediatria, Universidade Federal de São Paulo (UNIFESP), São Paulo, Brasil; 4 Instituto de Matemática e Estatística (IME), Universidade de São Paulo (USP), São Paulo, Brasil; 5 Biometric Research Branch (BRB), Division of Cancer Treatment and Diagnosis (DCTD), National Cancer Institute (NCI), National Institutes if Health (NIH), Bethesda, Maryland, USA; 6 "Ghost Lab", T-Cell Tolerance and Memory Section (TCTMS), Laboratory of Cellular and Molecular Immunology (LCMI), National Institute of Allergy and Infectious Disease (NIAID), National Institutes if Health (NIH), Bethesda, Maryland, USA

## Abstract

**Background:**

DAPfinder and DAPview are novel BRB-ArrayTools plug-ins to construct gene coexpression networks and identify significant differences in pairwise gene-gene coexpression between two phenotypes.

**Results:**

Each significant difference in gene-gene association represents a Differentially Associated Pair (DAP). Our tools include several choices of filtering methods, gene-gene association metrics, statistical testing methods and multiple comparison adjustments. Network results are easily displayed in Cytoscape. Analyses of glioma experiments and microarray simulations demonstrate the utility of these tools.

**Conclusions:**

DAPfinder is a new friendly-user tool for reconstruction and comparison of biological networks.

## Background

Microarray researchers need easy-to-use tools to identify differences in the coexpression and coregulation of genes between phenotypes that cannot be identified with traditional tools. Often researchers compute Student's t-tests, analysis of variance (ANOVA), significance analysis of microarrays [1] or empirical Bayes analysis [2] for each gene on their microarray to identify individual differentially expressed genes (DEGs) among two or more phenotypes [3]. Unfortunately, these approaches ignore coexpression because they cannot account for the complex multivariate relationships among genes. Multivariate statistical methods like hierarchical clustering and principle components analysis (PCA) are often used for quality control and exploration of microarray data. However, these multivariate methods do not effectively model coexpression nor do they allow for hypothesis tests to compare phenotypes. Gene-gene association networks built using ARACNe [4], context likelihood relatedness (CLR) [5], maximum relevancy (MR) [6,7] and other methods often provide helpful models of coexpression and coregulation, but the networks are based on data from a single phenotype and are not easily compared using statistical tests. New methods are needed to account for the complex relationships among genes while providing hypothesis tests to compare phenotypes.

Several research groups have addressed the question of comparing the coexpression of specific gene-gene pairs or coexpression networks among two or more phenotypes. Two early examples used search algorithms to identify optimally sized clusters of coexpressed genes and resampling tests to identify significant differences among the coexpressed clusters between phenotypes [8,9]. Other published methods used variations on familiar statistical techniques like Fisher's Z tests or modified F-statistics to directly compare pairwise gene-gene correlations between two phenotypes [10-12]. Some of these methods [10,11,13] are readily available as source scripts of package libraries in R http://www.r-project.org. Some interesting approaches apply the results from statistical tests that compare pairwise gene-gene associations between two phenotypes to the construction and interpretation of gene coexpression networks [10,14]. Both of these methods allow researchers to explore the complex differences among gene expression networks using statistical tests, but unfortunately neither method has been implemented in a user-friendly tool.

DAPfinder and DAPview are plug-ins for BRB-ArrayTools http://linus.nci.nih.gov/BRB-ArrayTools.html, which will provide researchers with accessible tools to test differences in the coexpression between two phenotypes and explore those results on gene association networks. BRB-ArrayTools is a comprehensive microarray analysis package that does not require specific skills in programming or direct script usage. It is available for free to non-commercial users and has more than 11,000 users in 65 countries [15]. Our DAPfinder and DAPview tools will identify and visualize individual significant differences in gene-gene association between the two classes, each of which we will call a Differentially Associated Pair (DAP). Output from these tools can be used to construct gene-gene association networks and identify the significant differences in coexpression between two groups. Our hope is that these tools can be used to identify systems-level features in the gene-gene association networks like network growth or decay, network merging or splitting, and network birth or death, reflecting functional changes in biological pathways.

## Implementation

### DAPfinder

DAPfinder is used to compute pair-wise gene-gene associations (i.e. gene-gene correlations) for two groups of microarray experiments, then compare each specific gene-gene association between the two groups with a statistical test (Additional file 1, Figure S1). Gene-gene associations can be estimated using Pearson correlation coefficients, Spearman rank correlation coefficients, Kendall rank correlation coefficients or mutual information. Pearson correlations are the most familiar metric and the easiest to compute, but only the Spearman, Kendall and mutual information metrics are appropriate for nonlinear associations between genes. Significant Pearson correlations within each class are identified using a one-sample Fisher's Z-test. Differences in gene-gene correlations (i.e. Pearson, Spearman and Kendall) are automatically tested using Fisher's Z-test methods, while optional permutation tests are used to compare differences in gene-gene correlation or mutual information. P-values from the Fisher's Z-test methods are approximate p-values that assume large sample sizes; permutation tests make no assumption about sample size, but they require lengthy computation times. Permutation test calculations can be hastened by choosing from one of four gene-gene pair subset selection methods (Additional file 1, Figure S1). Tests can be computed with equal numbers of permutations for each gene-gene pair or with an adaptive method that identifies the minimum number of permutations required for each gene-gene pair. Fisher's Z-tests of individual Pearson correlations within each class or differences in correlation between the two classes can be corrected for multiple testing using false discovery rate (FDR) methods [16,17], q-value methods [18-20] or Bonferroni family-wise error rate (FWER) methods using step-up adjusted p-values [21]. The same multiple testing adjustments can be applied to the optional permutation tests. Researchers can pre-filter individual genes by the coefficient of variation (CV) of their gene expression, by a minimum sample size criteria (after outliers and missing data have been removed) or using the internal methods of BRB-ArrayTools. Researchers can also upload a specific list of gene-gene pairs for testing. Outliers among the individual expression values from each gene can be removed using univariate standard deviation or interquartile range (IQR) criteria.

Output from DAPfinder includes a hyper-text markup language (HTML) report and comprehensive output stored as an Excel spreadsheet or tab-delimited text file. The HTML report opens up automatically in a web browser to display the current user settings and diagnostics from the analyses. Reported user settings include choices of pre-filtering methods, association metrics and statistical tests, plus the directory location of the results. Diagnostics include the amount of missing data, the number of genes and gene-gene pairs used in the calculations and the computation time required. Optionally, the 10 most significant results from the Fisher's Z-tests and permutation test can be added to the HTML report. The comprehensive output includes the unique IDs and related annotations for both genes in each gene-gene pair, the individual gene-gene associations for each of the two groups with test statistics and p-values reported for the Pearson correlations in each group, the Fisher's Z-test statistics and p-values for comparisons between the two groups and finally the differences in association and permutation p-values between the two groups (if requested). These results can be sorted and reorganized in Excel to identify the most significant gene-gene associations in a single group, the most significant Fisher's Z-test results, etc. Results from the comprehensive output file can be directly imported into visualization software packages like Cytoscape [[22], http://www.cytoscape.org] to create network graphs using the two columns of unique IDs to define nodes and the columns of correlation coefficients or p-values to define edge weights. Both the HTML report and the comprehensive output are automatically saved to the user's BRB-ArrayTools project folder.

### DAPview

**Figure 1:**
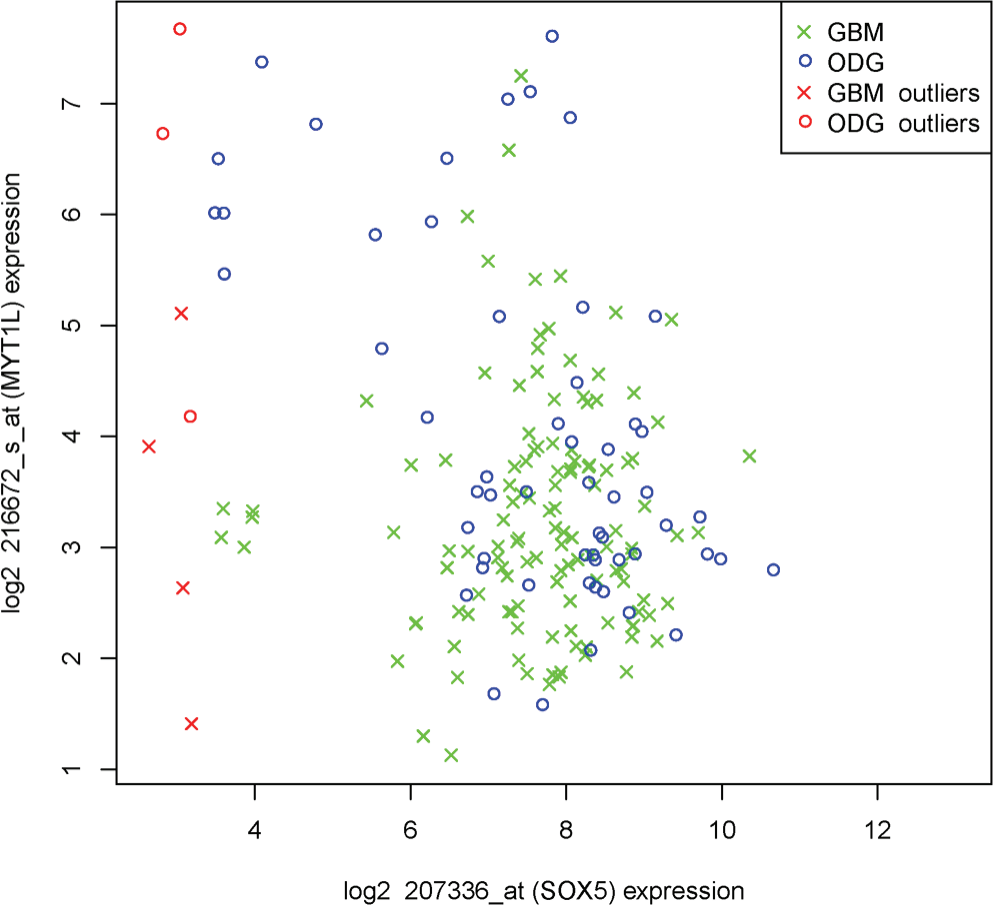
**Negative correlation between MYTL1 gene (probe set 216672_at) and SOX5 gene (probe set 207336_at) in oligodendrogliomas (ODG) and no association between SOX5 and MYTL1 in glioblastoma multiforme (GBM) illustrated using the DAPview**.

DAPview graphs the expression values for two specific genes in a XY scatter plot with the differences in coexpression between two phenotypes displayed in different colors and symbols (Figure 1). Typically, a statistically significant difference in gene-gene association would be discovered using DAPfinder, then the relationship can be visualized with DAPview. The two groups are graphed using different colors and symbols with a figure legend clearly identifying each group. Researchers can choose to identify, eliminate or ignore the outlier expression values identified by the same univariate standard deviation or interquartile (IQR) range criteria from DAPfinder. Identified outliers are plotted in red, while eliminated outliers are completely removed from the graph and ignored outliers are plotted in the same colors as the legitimate data. Scatter plot graphs are automatically opened in portable document file (PDF) format and saved into the user's BRB-ArrayTools project folder.

## Results

### Evaluation of DAPfinder with Simulated Microarray Data

The efficacy of the DAPfinder procedures was evaluated using simulated microarray data with known gene-gene correlations to ensure its statistical methods can detect known differences in gene-gene association with high levels of statistical power and low levels of false positives. See the supplementary materials (Additional file 1) for details on the generation of simulated microarray data and other simulation methods. Simulation results were used to create receiver-operator characteristic (ROC) curves that explore the relationships between statistical power, sample size and effect strength under several different simulation conditions. Other simulations examined the relationship between approximate p-values from the Fisher's Z-tests and exact p-values from the permutation tests. Simulations were conducted entirely in R using the same R source code used to build DAPfinder.

**Figure 2:**
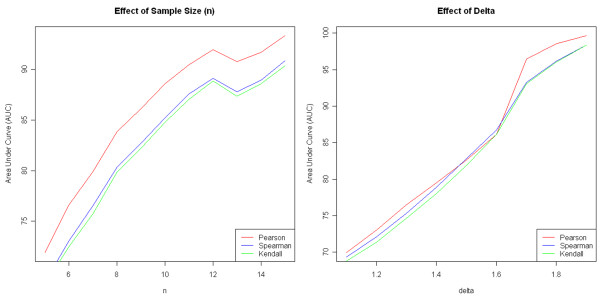
**Effects of sample size and and difference in correlation**. **Left**. Effect of increasing sample size on ROC AUC with 40 genes per chip, 250 simulation runs and constant delta = Δ*r *= *r_i _*- *r_j _*= +0.5 - (-0.5) = 1. **Right**. Effect of increasing difference in correlation between classes from delta = *r_i _*- *r_j _*= +0.55 - (-0.55) = 1.1 to *r_i _*- *r_j _*= +0.95 - (-0.95) = 1.9 on ROC AUC with 40 genes per chip, 250 simulation runs and constant sample size n = 5 chips per class.

**Figure 3:**
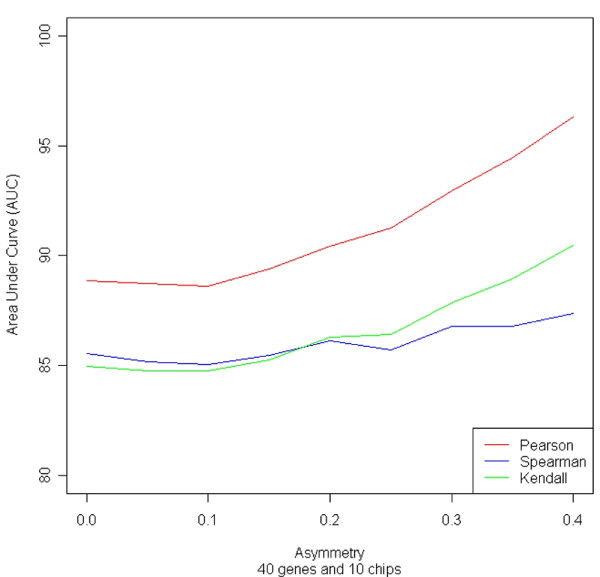
**Effect of increasing asymmetry on ROC AUC.** Asymmetry was increased from *r_i _*- *r_j _*= +0.5 - (-0.5) = 1) to *r_i _*- *r_j _*= +0.95 - (-0.05) = 1 with 40 genes per chip, 250 simulation runs and constant sample size n = 10 chips per class.

Examining changes in Area Under Curve (AUC) for the ROC quickly revealed many properties of the analyses in DAPfinder. Not surprisingly, results from the simulations show that sensitivity (i.e. statistical power) and specificity (i.e. control over false positives) increase as sample sizes (*n*) or differences in correlation (delta = Δ*r *= *r_i _*- *r_j_*) increase (Figure 2) when all other experimental conditions are held constant (Additional file 1, supplementary information). These results show the DAPfinder performs well even for relatively small sample sizes and differences in correlation. Increasing as the number of genes on each microarray chip did not affect sensitivity or specificity (Additional file 1, Figure S6), supporting our decision to use simulations with a small number of genes per chip because they are more efficient (see Additional file 1, supplementary materials, for details). In the real world, increasing the number of genes per chip would decrease statistical power due to the more conservative FDR- and FWER adjustments for multiple testing and possible due to higher level interactions among large numbers of genes. However, these simulations computed ROC curves using unadjusted p-values and fixed numbers of interacting genes. It may be surprising that sensitivity and specificity did not change as the expression variances of individual genes increased (Additional file 1, Figure S7) with all other experimental conditions held constant. However, increasing the individual gene expression variance does not affect sensitivity and specificity, because the correlation of two genes is a property of the joint distribution that is not solely dependent on the magnitude of individual gene expression variances. Perhaps the most important simulation result showed that sensitivity and specificity increased as correlation coefficients from the two groups changes from perfectly symmetric with *r_i _*- *r_j _*= +0.5 - (-0.5) = 1 to increasingly asymmetric coefficients like *r_i _*- *r_j _*= +0.95 - (-0.05) = 1 or *r_i _*- *r_j _*= +0.05 - (-0.95) = 1 with all other conditions held constant (Figure 3). Asymmetric correlation coefficients have more statistical power because the nonlinear Fisher's Z-transformation used in the Fisher's Z-test inflates z-scores for strong correlations and deflates z-scores for moderate correlations (Additional file 1, Figure S8), creating larger differences in Z-scores and more significant Fisher's Z-tests.

**Figure 4:**
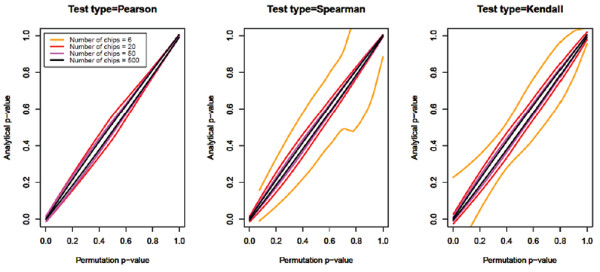
**Effect of increasing sample size on the relationship between analytical (i.e. approximate) p-values from Fisher's Z-test procedures and permutation (i.e. exact) p-values**.

Additional simulations showed approximate p-values from the Fisher's Z-test of differences in Pearson correlation are strongly correlated to the exact p-values from the permutation tests, and the correlation between the approximate and exact p-values increases with sample size (Additional file 1, Figure S9). Similar correlations between approximate p-values and exact p-values are seen for differences in Spearman rank correlation and differences in Kendall rank correlations (Figure 4). These show the robust results from computationally intensive permutation and resampling tests can be very closely approximated by much faster Fisher's Z-test and similar methods for Spearman and Kendall rank correlations with reasonable sample sizes. Both options are included to provide researchers the option of faster computation when sample sizes are relatively large or more robust results when sample sizes are smaller.

### Discoveries from Glioma Data

**Figure 5:**
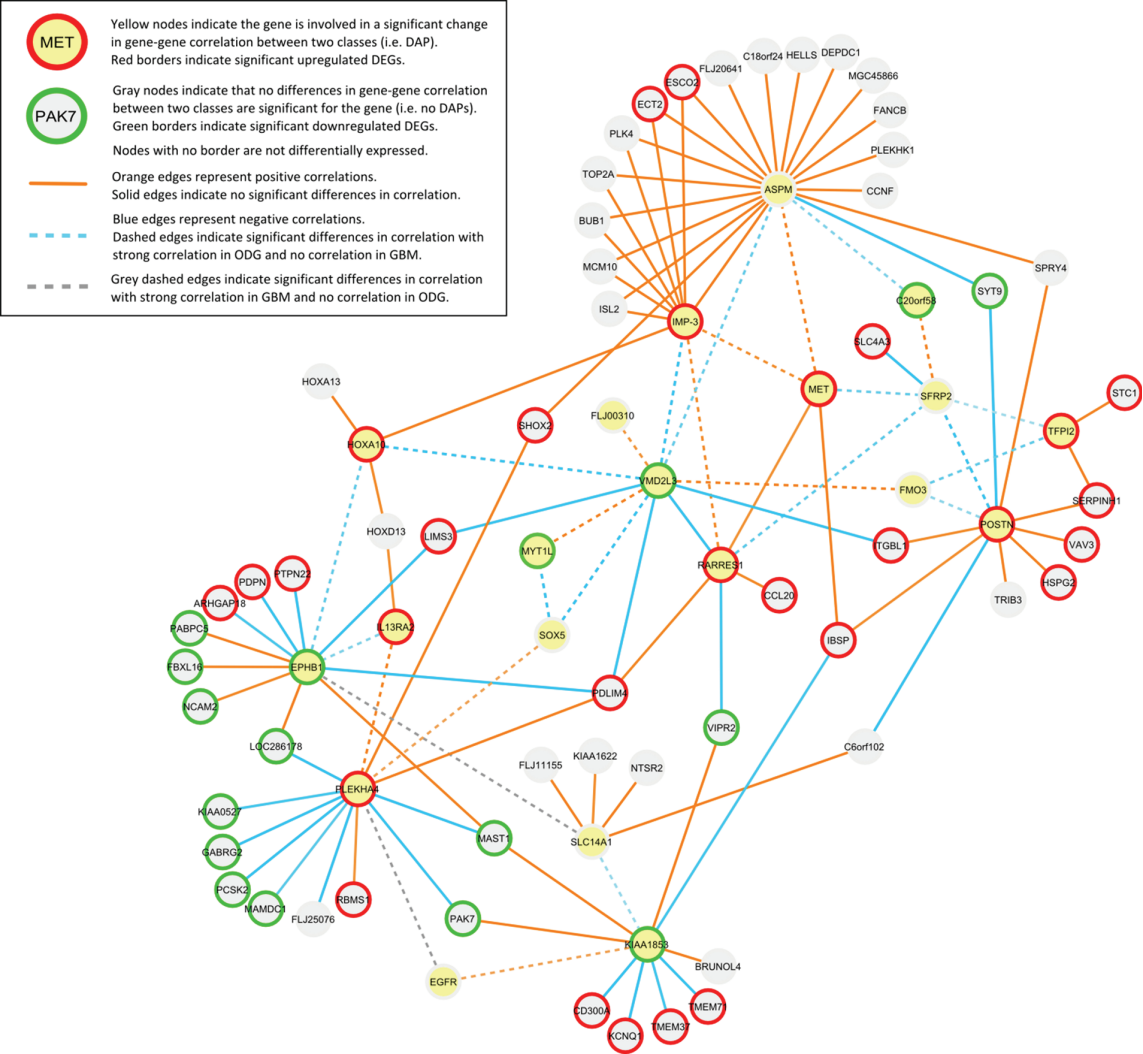
**A gene-gene association network created in Cytoscape using output from DAPfinder**. Nodes with yellow fill identify genes involved in statistically significant DAPs. Nodes with gray fill identify genes that were not involved in any significant differences in gene-gene correlation between ODG and GBM tumors, but consistently correlate with the "yellow" genes in both ODG and GBM. Nodes with red borders indicate that a gene was upregulated in ODG, nodes with green borders represent genes that were downregulated in ODG and nodes with no border did not have any significant differential expression. Orange edges represent positive correlations between two genes in the ODG tumors. Blue edges denote negative correlations between genes in ODG tumors. Gray edges represent strong correlations in the GBM tumors, but no correlation in ODG tumors. Solid edges represent correlations that are consistent in both ODG and GBM tumors, while dashed edges represent statistically significant DAPs where correlations are not consistent among ODG and GBM groups.

To illustrate some possible uses of the DAPfinder, we analyzed transcriptional data from glioma samples publicly available in the Repository of Molecular Brain Neoplasia Data (REMBRANDT) [[23], http://caintegrator.nci.nih.gov/rembrandt]. We used data from oligodendroglioma (ODG) and glioblastoma multiforme (GBM) samples representing low and high malignancy primary adult brain tumors, respectively [24]. We identified significant differences in Pearson correlation (p < 0.10 and *r_i _*- *r_j _*> 0.50) between ODG and GBM tumors for 727 gene-gene pairs which were consistent in tumors from two independent studies at Henry Ford Hospital [25] and the Glioma Molecular Diagnostics Initiative (GMDI) [26]. We constructed a gene-gene association network by focusing on a cluster of 27 gene-gene pairs (from 20 genes) with significant differences in Pearson correlation between ODG and GBM tumors and an additional 85 gene-gene pairs (from 56 genes) that are connected to this cluster of 27 DAPs by correlations of similar strength and direction in both classes of gliomas (Figure 5). See supporting materials for details.

We noticed three features in the network that were not necessarily expected. First, more than half of the genes from this network were differentially expressed between the two classes of glioma (46 out 76 genes). This suggests there may be a general correlation between differential expression and differences in association between phenotypes. Second, the relationship between differential expression and direction of correlation from consistent edges may represent potential regulatory relationships among genes. Positive correlations occur whenever both genes are up- or down-regulated, while negative correlations occur whenever one gene is up-regulated and the other is down-regulated. Note, because the correlations are estimated within the same type of samples, either ODG or GBM, the fact that genes are up- or down-regulated in GBM relative to ODG should not influence the correlation results. This phenomenon is seen in all 48 correlations that are consistent between the ODG and GBM tumors. Third, the significant differences in gene-gene association seem to reflect the biological differences between ODG and GBM. Correlations that change direction between glioma types typically show strong positive or negative correlations consistent with regulation in ODG, while having zero correlation in GBM. This suggests that evolution of the tumor may lead to the loss of regulatory relationships in the de-differentiating tissue. The gene-gene association shrinks from 76 genes and 110 gene-gene pairs in ODG to 69 genes and 87 gene-gene pairs in GBM, suggesting systems-level network shrinkage from ODG to GBM resulting in loss of regulation functions.

Among the significant correlation changes in the network, we find three genes (MYT1L, EGFR, POSTN) known to have meaningful roles in glioma pathogenesis [27-29]. Myelin transcription factor 1 (MYTL1) is upregulated in the less malignant ODG tumors and it is a major factor necessary for neuronal differentiation [30]. The significant difference in Pearson correlation between SOX5 and MYTL1 in ODG and GBM tumors is visualized with DAPview (Figure 1). Epidermal growth factor receptor (EGFR) is a famous member of the erbB family of receptors that involved in regulation of cell proliferation and differentiation. Deregulation of EGFR was shown to have critical role in gliomas [31] as well as in several other malignancies [32-36]. Up-regulation in the protein-coding gene POSTN (periostin) is correlated with metastasis in both melanoma and breast cancer [37]. Although this analysis does not allow for definitive biological conclusions, it finds both previously established genes essential for tumorgenesis as wells as points to a new previously unexplored area of transcriptional regulation of gliomas. These results support the idea that estimating not only the structure but also changes in the co-expression gene networks can be a useful approach for understanding the disease process.

## Conclusions

Analyses of empirical and simulated microarray data have shown that DAPfinder is a powerful tool to reconstruct and compare gene regulatory networks. Its design is not restricted to gene expression data from single channel and dual channel microarray experiments. The tool can also be used with expression data from RNA-Seq reads or it can analyze complex quantitative biological data like comparative genomic hybridization (CGH), metabolome, microbiome and proteome data. DAPfinder can also be used to compute gene-gene associations and construct gene coexpression networks, even when there is not a second phenotype for comparisons of gene-gene associations and networks. DAPfinder can be used within BRB-ArrayTools by biologists without specific skills in programming and/or direct script usage. Indeed, we have recently employed the tool in the meta-analysis of cervical cancer gene expression and comparative genomic hybridization data revealing critical events of tumor progression (Mine KL, Shulzhenko N, Yambartsev A, *et al*.: Reconstruction of an integrative gene regulatory meta-network reveals cell cycle and antiviral response as major drivers of cervical cancer, submitted). Future versions may extend the utility of the statistical tests and graphs to problems with 3 or more phenotypes, while alternative gene-gene association metrics and statistical tests can also be explored to ensure proper networks construction.

## Availability and requirements

DAPfinder and DAPview may be downloaded for free from the NIAID Exon website http://exon.niaid.nih.gov/dapfinder/index.html. Complete installation instructions are provided on the website. DAPfinder and DAPview requires the installation of BRB-ArrayTools. BRB-ArrayTools currently requires the installation of Microsoft Excel, Java Virtual Machine, R 2.12.0 or higher and statconnDCOM on computer using the Microsoft Windows operating system. DAPfinder and DAPview are BRB-ArrayTools plug-ins, which mostly utilize open source R script files. A complete description of the DAPfinder and DAPview files can be found in our supplementary materials (Additional file 1). DAPfinder and DAPview are also available to download as Additional Files 2 and 3.

## List of abbreviations

ANOVA: Analysis of Variance; ARACNe: Algorithm for the Reconstruction of Accurate Cellular Networks; AUC: Area Under Curve; CGH: Comparative Genomic Hybridization; CLR: Context Likelihood of Relatedness; CV: Coefficient of Variation; DAP: Differentially Associated Pair; DEG: Differentially Expressed Gene; FDR: False Discovery Rate; FWER: Family-Wise Error Rate; GBM: Glioblastoma multiforme; GMDI: Glioma Molecular Diagnostics Initiative; HTML: Hyper Text Markup Language; IQR: Interquartile Range; MR: Maximum Relatedness or Minimum Redundancy; ODG: Oligodendroglioma; PCA: Principle Components Analysis; PDF: Portable Document File; REMBRANDT: Repository of Molecular Brain Neoplasia Data; ROC: Receiver Operator Characteristic.

## Competing interests

The authors declare that they have no competing interests.

## Authors' contributions

JS was the primary software developer, critically contributed to the overall concept of the software, performed and analyzed the simulation experiments, and drafted the manuscript. YK analyzed the glioma data and simulation results, drafted part of the manuscript related to the glioma data and provided input on the development of the software. SV assisted with software development, contributed the adaptive permutation test feature to the software, performed and analyzed simulation experiments. KLM tested the software and provided input on the development of the software. AY conceived the original idea for the software, provided input on the development of the software and assisted with the generation of simulated microarray data. RS provided formulas for approximate tests of Kendall and Spearman correlations, contributed the single class correlation selection procedure to select gene-gene pairs for permutation tests. YH supervised the software development project, provided web distribution of the software and provided input on the development of the software. AM conceived the original idea for the software, provided the original specifications for the software, analyzed and interpreted glioma data and simulation results, drafted parts of the manuscript and provided scientific leadership of the project. All authors reviewed and approved the manuscript.

## Authors' information

None.

## Supplementary Material

Additional file 1 **Additional information and Supplemental figures not included in the article**. Additional Details About DAPfinder Methods; Development details of DAPfinder and DAPview; Validation of DAPfinder with Simulated Microarray Data; Discoveries from Glioma Data. Click here for file

Additional file 2 **DAPfinder**. DAPfinder plug-in software for BRB-ArrayTools. Click here for file

Additional file 3 **DAPview**. DAPview plug-in software for BRB-ArrayTools. Click here for file
