# Supplementary material for: Construct and Compare Gene Coexpression Networks with DAPfinder and DAPview
Source: BMC Bioinformatics. 2011 Jul 14;12:286. doi: 10.1186/1471-2105-12-286 (PMC3149583; doi:10.1186/1471-2105-12-286)
Supplement: Additional file 1 — Additional information and Supplemental figures not included in the article. Additional Details About DAPfinder Methods; Development details of DAPfinder and DAPview; Validation of DAPfinder with Simulated Microarray Data; Discoveries from Glioma Data. [file 1471-2105-12-286-S1.PDF]

Supplementary Materials

Additional Details About DAPfinder Methods

The DAPfinder plug-in allows users to filter their gene expression data, compute gene-gene associations using one of four different metrics, compare gene-gene associations using Fisher's Z test or permutation test methods and finally compute multiple comparison adjustments for the p-values using one of four different methods (Additional file 1, Figure S1).

The formula for a two-sample Fisher's Z-test to compare Pearson correlations between two phenotypes is well known:

$$z_i = \frac{1}{2} ln \frac{1+r_i}{1-r_i} = arctanh(r_i)$$

$$F^*_{Pearson} = \frac{z_i - z_j}{\sqrt{\frac{1}{n_i - 3} + \frac{1}{n_j - 3}}}$$

Formulas for two-sample Fisher's Z-tests to compare Spearman rank correlations and Kendall's Tau rank correlations may be less familiar:

$$F^*_{Spearman} = \frac{sp_i - sp_j}{\sqrt{\frac{1}{n_i - 1} + \frac{1}{n_j - 1}}}$$

$$F^*_{Kendall} = \frac{kn_i - kn_j}{\sqrt{2\frac{(2n_i + 5)}{9n_i(n_i - 1)} + 2\frac{(2n_j + 5)}{9n_j(n_j - 1)}}}$$

Permutation test options include four different methods to select a subset of gene-gene pairs for permutation testing (Additional file 1, Figure S2) and two different options to determine the appropriate number of permutations to test each gene-gene pair. Subset selection methods choose gene-gene pairs for permutation testing by either identifying the largest differences in association between the two classes or by identifying the most significant correlations in a single class. Users can choose to compute an equal number of permutations for all gene-gene pairs, or an adaptive permutation test method can be used. The adaptive permutation test method computes a block of 100 permutations for each gene, then constructs a confidence interval around the current permutation test p-value using the binomial distribution. The confidence interval indicates whether the current permutation test p-value is "clearly significant", "clearly non-significant" or whether it requires further testing. For example, if the p-value cut-off value is p = 0.05 and a 95% confidence interval indicates the current permutation p-value is between 0.000 and 0.013, then the current p-value is "clearly significant". If the 95% confidence interval indicates the current permutation p-value is between 0.462

and 0.599, then the current p-value is “clearly non-significant”. If 95% confidence interval is between 0.034 and 0.075, then further testing is needed and another block of 100 permutations is computed. A second confidence interval is computed from all 200 permutations and the process is repeated until all p-values are “clearly significant” or “clearly non-significant”.

Development of DAPfinder and DAPview

DAPfinder and DAPview were developed using the BRB-ArrayTools plug-in utilities, R source script files and one binary file written in C. BRB-ArrayTools allows users to generate a new plug-in from at least one R source script using a point-and-click menu system which creates a .plug file for each plug in. The DAPfinder.plug file links BRB-ArrayTools to the initial R source script file and generates one pop-up window dialogue with buttons and text box inputs. The initial R script loads additional R source files and one C binary file. The DAPfinder.R source script is called by the DAPfinder.plug file and organizes all of the steps of the analysis. The functions.R source file stores most of the novel R functions created for this workflow. The perm.all.R source file contains the novel R functions related to the permutation tests. The permute_gene.dll C binary is called by the perm.all.R source file and performs many of the permutation test computations to speed up computations in DAPfinder. The dialogs.R source file defines all of the interactive menus for the graphic user interface (GUI) using tcl/tk. Additional files distributed with DAPfinder include a DAPfinder.txt help file and a PDF instruction manual.

The R source files depend on 7 R package libraries. The *gtools* R package library is required, so its *permute* function can be used in the permutation tests. The *minet* R package library is required for several functions used in the mutual information calculations. The *psych* R package library is required for its *fisherz* function used to compute Fisher’s Z-test for Pearson correlations. The *qvalue* package library is required for the q-value computations for multiple comparison adjustments. The *R2HTML* R package library is required to simplify the production of the HTML report file. The *tcltk* R package library is required to produce the interactive GUI elements for DAPfinder.

Validation of DAPfinder with Simulated Microarray Data

Simulated microarray data were generated from pseudo-random samples of a multivariate normal distribution with mean $\boldsymbol{\theta}$ and variance-covariance matrix Σ using the *mvtnorm* package library in R. The mean parameter $\boldsymbol{\theta}$ was generated from a pseudo-random sample of the univariate normal distribution using the *stats* package library in R. The variance-covariance matrix Σ was generated in two steps. First, the expression variances of each simulated gene were generated from pseudo-random samples of the inverse gamma distribution (with parameters shape = 3 and scale = 1 unless specified), and these individual gene expression variances were stored in the diagonal elements of Σ. The distributions of individual gene expression variances σ_i^2 from real microarray experiments often closely match an inverse gamma distribution with similar parameter values. Second, known pair-wise gene-gene correlations were added to the off-diagonal elements of Σ using the formula $cov_{i,j} = \rho\sigma_i\sigma_j$ for $i \neq j$,

where ρ is a known correlation constant and σ_i , σ_j are the gene expression standard deviations of two genes as defined in step 1. These known pair-wise gene-gene correlations were added in blocks of 4 correlated genes separated by 1 independent gene each (Additional file 1, Figures S3 and S4). Blocks of correlated genes were arranged in the form of a complete graph (Additional file 1, Figure S3), because this design ensures that correlations from the pseudo-random sample have the desired correlation strengths. For example, if desired correlations of +0.75 and -0.75 are specified using a complete graph design, the observed correlations from a specific pseudo-random sample might be +0.743 and -0.769. However, if the desired correlations of +0.75 and -0.75 are specified in a naïve design, the observed correlations from a specific pseudo-random sample might be + 0.515 and -0.488 or weaker. Using this complete graph arrangement also seems to ensure the variance-covariance matrix will be defined as a positive semi-definite matrix, which is a necessary condition of all variance-covariance matrices.

Pseudo-random samples from this multivariate normal distribution could be drawn with different numbers of simulated genes per chip (g) and different numbers of simulated microarray chips per group (n_1 and n_2). Correlation strength (ρ) was held constant between all correlated gene-gene pairs in a single simulation experiment, but the correlation strength could be varied from one simulation experiment to another. A known proportion of the individual expression values from simulated data could be deleted to simulate missing data or replaced with a pseudo-random sample from the Cauchy distribution to simulate outlier observations, if desired. The resulting data should closely simulate a sample of real microarray data after $\log_2$-transformation and other typical processing steps.

Each simulation experiment would generate hundreds of simulated data sets to test the DAPfinder computations. The R source files from DAPfinder were used to compute differences in Pearson correlation, Spearman rank correlation and Kendall rank correlation with all their related p-values for each simulated data set. True positive and false positive rates were computed for each simulated data set, using the fact that the identities of all of the correlated and uncorrelated gene-gene pairs were defined during the simulation process, then the true positive and false positive rates from each data set were averaged together over all of the hundreds of data sets in the simulation experiment. True positive and false positive rates were computed for every possible p-value cut-off from p = 0.00 to p = 1.00 so ROC curves could be generated under multiple experimental conditions, while AUC was computed for each ROC curve using the trapezoid method to quickly summarize these relationships.

ROC curves were always computed using unadjusted p-values, because FDR- or FWER-adjusted p-values produce uninformative ROC curves and misleading AUC values (Additional file 1, Figure S5) due to the fact that the adjustments put strong constraints on the false positive rate (specificity). Counter-intuitively, applying FDR- or FWER-adjustments always raises ROC curve AUC, even though they strongly reduce the sensitivity of the tests. This is because the strong control of false positive rate creates a strong, almost linear slope near false positive rate = 0 on the ROC curve. This creates ROC curves that are uninformative or misleading.

The effect of increasing sample size on ROC AUC was explored by holding all other simulation conditions constant. Specifically, if you hold delta constant ($r_i - r_j$ = +0.5 – (-0.5) = 1) with 40 genes per

chip and 250 simulation runs, then AUC of the ROC curves from Pearson correlation increased from 71.9% to 93.35% as sample size increased from n = 5 to n = 15 simulated microarray chips per group (Figure 2, left). Under the same conditions, as sample size increased from n = 5 to n = 15 per group, the AUC of the ROC curves from Spearman and Kendall rank correlation increased from 89.14% and 88.86% to 90.85% and 90.38%, respectively.

The effect of increasing the magnitude of the difference in correlation between two groups was examined by holding other conditions constant. Specifically, sample size was constant (n = 5 chips per class) with 40 genes per chip and 250 simulation runs, then AUC of the ROC curves from Pearson correlation will increase from 69.92% to 99.68% as delta increased from $r_i - r_j$ = +0.55 – (-0.55) = 1.1 to $r_i - r_j$ = +0.95 – (-0.95) = 1.9 (Figure 2, right). Under the same conditions, as delta increased from $r_i - r_j$ = 1.1 to $r_i - r_j$ = 1.9, the AUC of the ROC curves from Spearman and Kendall rank correlation increased from 69.32% and 68.76% to 98.39% and 98.37%, respectively.

Increasing the number of genes per chip did not affect sensitivity and specificity (Additional file 1, Figure S6). Holding sample size constant at n = 10 chips per class and difference in correlation constant at $r_i - r_j$ = +0.5 – (-0.5) = 1, the AUC of the ROC curves from Pearson correlation changed from 89.05% to 88.5% as the number of genes per chip increased from g = 5 to g = 100, respectively. The simulations reached minimum AUC = 87.51% at g = 10 and maximum 89.13% at g = 25. This finding was important, because it was difficult to generate simulated microarray chips with realistically large numbers of genes per chip (e.g. 10,000-100,000 genes per chip). It was also inefficient to generate simulated microarray chips with large numbers of genes per chip, because correlated gene-gene pairs could only be created in small complete graphs on the main diagonal of the variance-covariance matrix of the multivariate normal distribution. Therefore, as the number of genes per chip increases, the relative proportion of known differences in gene-gene correlations decreases. For that reason, the rest of the simulations use a small number of genes per chip (e.g. g = 40) with a larger number of simulation runs (e.g. 250 runs), because it is more efficient than a simulation with a larger number of genes per chip (e.g. g = 2,500) and a smaller number of simulation runs (e.g. 4 runs).

Sensitivity and specificity did not change as distribution of individual gene expression variances changed to produce more genes with larger gene expression variances (Additional file 1, Figure S7 left). Holding sample size constant at 10 chips and difference in correlation constant at $r_i - r_j$ = +0.5 – (-0.5) = 1 with 40 genes per chips and 250 simulation runs, ROC curves were computed for 7 different combinations of inverse gamma distribution parameters: (1) shape = 1.0 and scale = 0.1, (2) shape = 1.5 and scale = 0.3, (3) shape = 3.0 and scale = 1.0, (4) shape = 8.0 and scale = 5.0, (5) shape = 10.0 and scale = 10.0, (6) shape = 12.0 and scale = 18.0 and (7) shape = 14.0 and scale = 30.0. As the parameter combinations increased from (1) to (7), the distribution of individual gene expression variances ranged from a tall, short tailed distribution mostly producing small gene expression variances (e.g. $\sigma^2 < 0.5$) to a flat, long tailed distribution mostly producing large gene expression variances (e.g. $\sigma^2 > 1.5$) (Additional file 1, Figure S7 right). These simulations produced a minimum AUC = 88.16%, 84.99% and 84.52% with a maximum AUC = 88.56%, 85.32% and 84.91% for Pearson, Spearman and Kendall, respectively (Additional file 1, Figure S7 left). Holding sample size constant at 10 chips and difference in correlation

constant at $r_i - r_j$ = +0.75 – (-0.75) = 1.5 with 40 genes per chips and 250 simulation runs, using the same 7 combinations of inverse gamma distribution parameters, the simulations produced a minimum AUC = 99.2%, 98.26% and 98.22% and a maximum AUC = 99.39%, 98.53% and 98.45% for Pearson, Spearman and Kendall, respectively (Additional file 1, Figure S7 left).

Sensitivity and specificity increased as correlation coefficients became more and more asymmetric (Figure 3). Specifically, if you hold sample size constant at 10 chips per group and delta constant at Δr = 1 with 40 genes per chip and 250 simulation runs, then AUC of the ROC curves from Pearson correlation increased from 88.86% to 97.95 as the correlation coefficients r_i and r_j changed from perfectly symmetric $r_i - r_j$ = +0.5 – (-0.5) = 1 to highly asymmetric $r_i - r_j$ = +0.95 – (-0.05) = +0.05 – (-0.95) = 1. This effect was less pronounced for Spearman and Kendall rank correlations, where AUC of the ROC curves increased from 85.55% and 84.97% to 88.85% and 93.03%, respectively, under the same conditions. The weaker effect on Spearman and Kendall rank correlations likely coincides with the information lost during the ranking procedure. The nonlinear Fisher's Z-transformation (Additional file 1, Figure S8) inflates z-scores for strong correlations (e.g. r = 0.95) and deflates z-scores for moderate correlations (e.g. r = 0.5). For example, with sample size n = 10 chips per class, the symmetric correlation coefficients -0.5 and +0.5 produce z-scores -0.5493 and + 0.5493, respectively, with a Fisher's Z-test statistic $F = (0.5493 - (-0.5493))/(1/(10 - 3) + 1/(10 - 3))^{1/2} = 2.0553$ and p = 0.0398. Alternatively, with n = 10 chips per class, the asymmetric correlation coefficients +0.95 and -0.05 produce z-scores +1.8318 and -0.0500, respectively, with a Fisher's Z-test statistic $F = (1.8318 - (-0.0500))/(1/(10 - 3) + 1/(10 - 3))^{1/2} = 3.5206$ and p = 0.0004.

The correlation between analytical (i.e. approximate) p-values from Fisher's Z-tests and exact permutation tests became stronger as sample sizes increased (Additional file 1, Figure S9). The same trend was observed for approximate tests of Spearman and Kendall rank correlations, using approximate testing procedures derived from Fisher's exact test. These simulations used 25 genes per chip with 500 simulation runs and delta = $r_i - r_j$ = 0 – 0 = 0. Note that delta = $r_i - r_j$ = 0 was used to show the correlation between asymptotic and exact p-values for all 0 < p < 1; if a stronger value of delta = $r_i - r_j$ was used, then the correlation between approximate and exact p-values would have been calculated over a much smaller range of p-values.

Discoveries from Glioma Data

We independently analyzed 187 ODG and GBM glioma tumors samples obtained at Henry Ford Hospital before 2004 (Sun *et al*. 2006) and the Glioma Molecular Diagnostics Initiative (GMDI) project (Li *et al*. 2009). There were 42 ODG samples and 76 GBM samples from Henry Ford Hospital; there were 17 ODG samples and 52 GBM samples from GMDI. Samples are downloadable as one single dataset, but sample IDs ("HF" and "GDMI") were retained to run separate analyses on each dataset. All specimens were profiled with Affymetrix HG-U133 Plus 2.0 expression arrays. Expression values were generated with the MAS5 algorithm using the standard CDF file.

Each dataset was analyzed separately with DAPfinder. Probe sets were filtered to remove any probe set without a gene symbol and only the 1000 genes with the highest variation were selected for

further analysis. After identifying the unique genes selected from each dataset, we reran the analyses with the complete list of 1,368 genes selected from both datasets. This list of 1,368 genes included 356 genes differentially expressed between ODG and GBM in the HF tumor samples and 232 genes differentially expressed between ODG and GBM in the GMDI tumor samples.

Pearson correlations were calculated for these selected gene-gene pairs after applying univariate outlier removal (outlier < $Q1 – 3*IQR_{pooled}$ or outlier > $Q3 + 3*IQR_{pooled}$). Fisher's Z-tests were used to evaluate differences in Pearson correlation between ODG and GBM for 1,273,579 gene-gene pairs in both datasets. There were 250,164 statistically significant ($p < 0.10$) differences in Pearson correlation among the two data sets (133,599 from HF; 130,712 from GMDI; and 14,147 common to both data sets). Only 727 of 14,147 statistically significant DAPs common to both data sets had a difference in Pearson correlation coefficient of delta = $r_i – r_j > 0.5$ (355 pairs) or delta < -0.5 (372 pairs). Only 13,306 of 133,599 significant DAPs from HF had delta = $r_i – r_j > 0.5$ or delta < -0.5, while 105,045 of 130,712 significant DAPs from GMDI had delta = $r_i – r_j > 0.5$ or delta < -0.5. If more than one probe set matched a unique gene symbol, then we selected the probe set with largest number of significant DAPs. Removing the redundant probe sets left 806 genes and 324,415 gene-gene pairs.

Sixty of these 806 genes were present in 2 or more gene-gene pairs with significant differences in Pearson correlation between ODG and GBM in both the HF and GDMI data sets. Those 60 genes form a total of 93 highly connected gene-gene pairs, with each pair representing a significant DAP in both data sets. We selected these 93 highly connected DAPs to build an initial gene-gene association network in Cytoscape. We selected the largest cluster (20 genes forming 27 gene-gene pairs) from this initial network to build our final network. From that cluster of 20 genes with significantly different correlation between 2 classes, we found 85 gene-gene pairs with correlations of the same direction and p-value < 0.05 in both the HF and GMDI data sets from an additional 56 genes. These 112 selected gene-gene pairs (27 pairs from the cluster of DAPs and 85 connected pairs) were visualized as the final network (Figure 5).

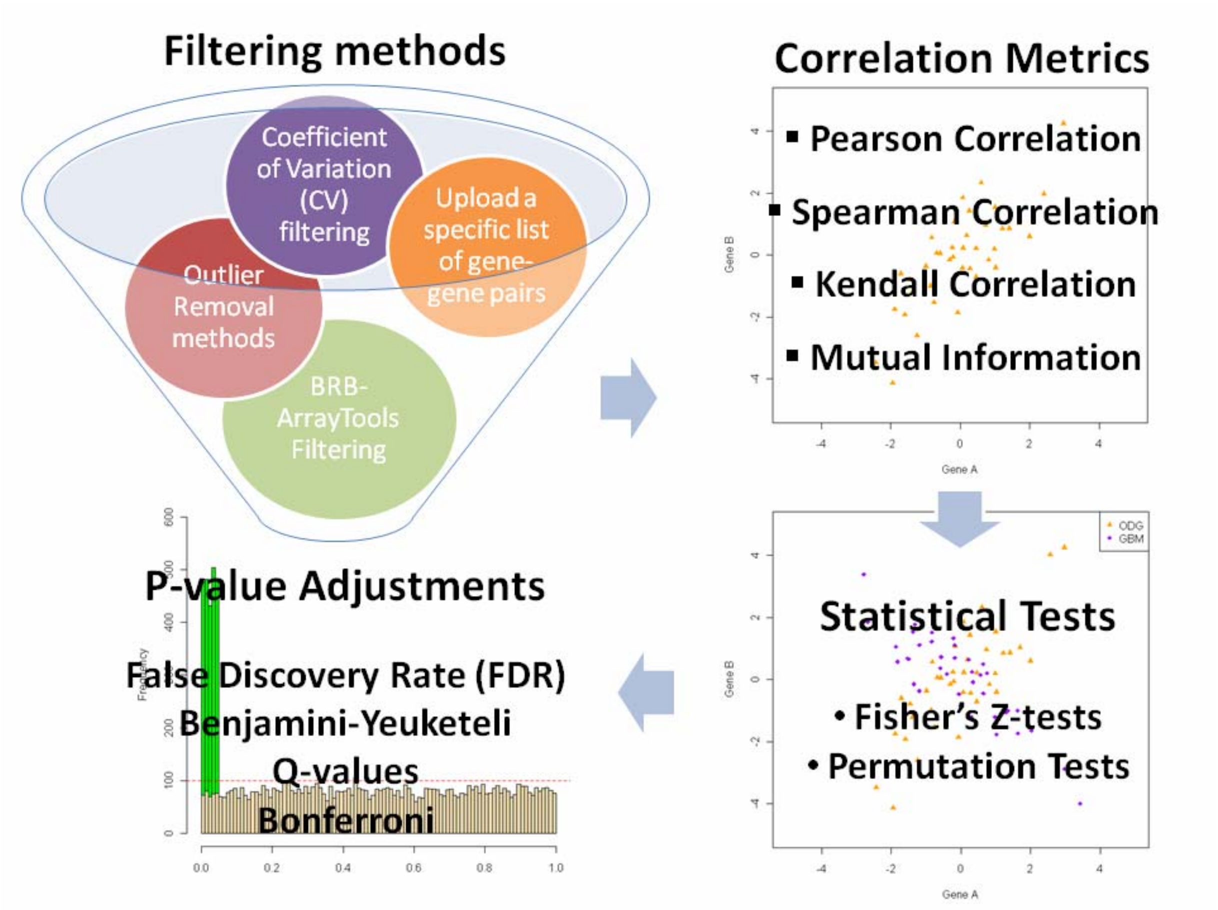

Figure S1. Flow chart of important DAPfinder features.

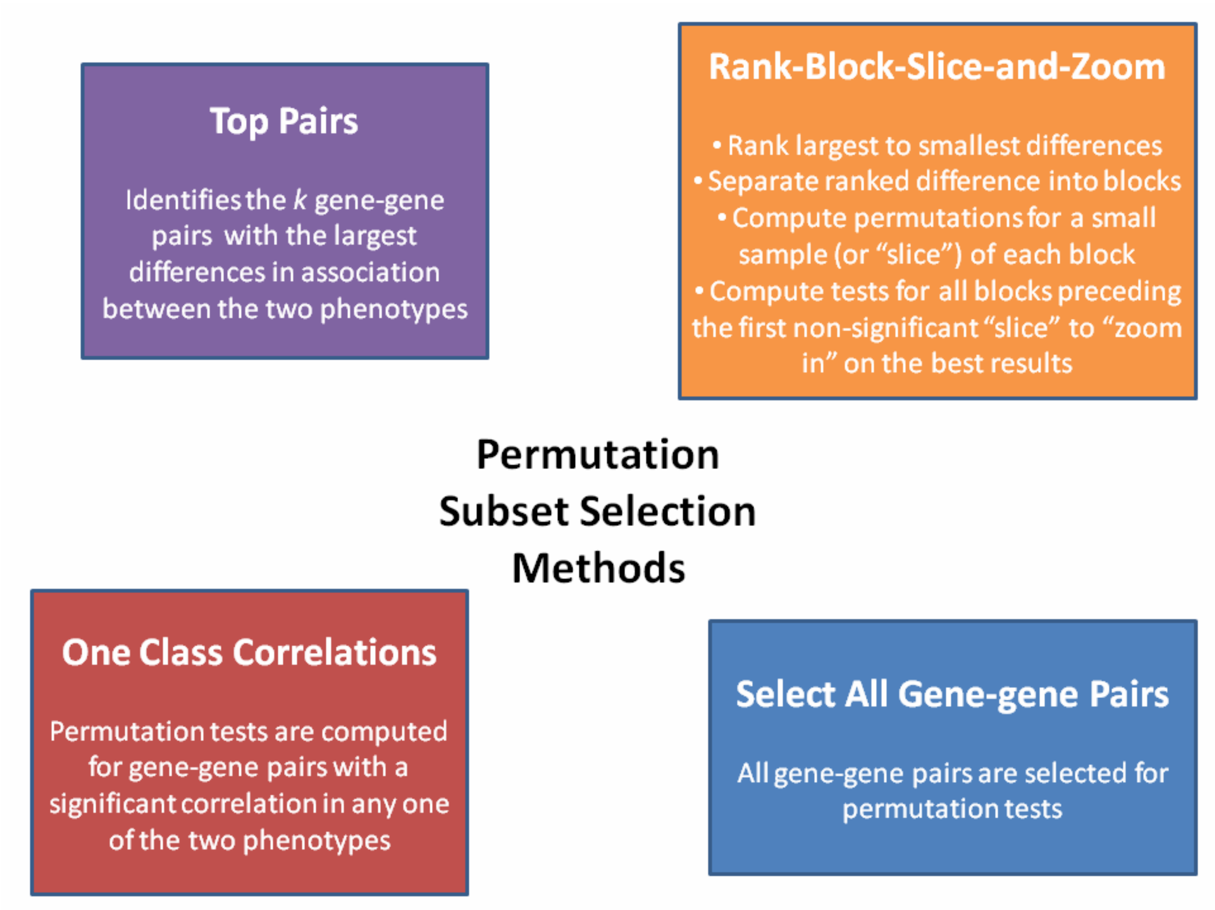

Figure S2. Description of permutation test sub-set selection methods.

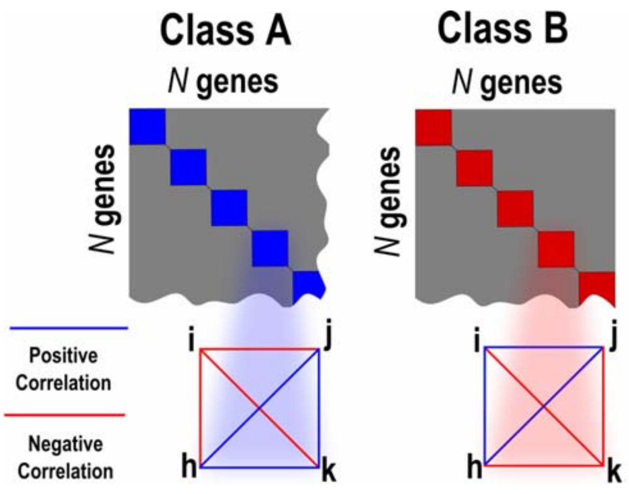

Figure S3. Arrangement of correlated gene-gene pairs in the variance-covariance matrix using to define simulated microarray data.

$$\begin{bmatrix} \sigma_1^2 & -\rho\sigma_1\sigma_2 & \rho\sigma_1\sigma_3 & \rho\sigma_1\sigma_4 & 0 & 0 \\ -\rho\sigma_1\sigma_2 & \sigma_2^2 & -\rho\sigma_2\sigma_3 & -\rho\sigma_2\sigma_4 & 0 & 0 \\ \rho\sigma_1\sigma_3 & -\rho\sigma_2\sigma_3 & \sigma_3^2 & \rho\sigma_3\sigma_4 & 0 & 0 \\ \rho\sigma_1\sigma_4 & -\rho\sigma_2\sigma_4 & \rho\sigma_3\sigma_4 & \sigma_4^2 & 0 & 0 \\ 0 & 0 & 0 & 0 & \sigma_5^2 & 0 \\ 0 & 0 & 0 & 0 & 0 & \ddots \end{bmatrix}$$

Figure S4. Variance-covariance matrix example.

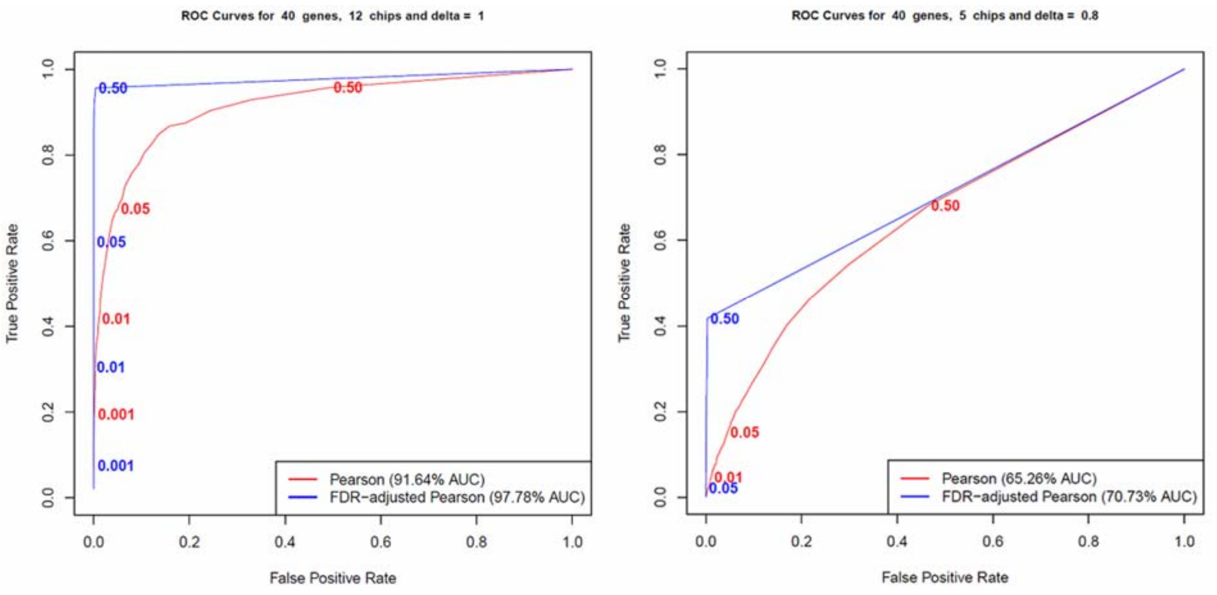

Figure S5. ROC curves of Pearson correlations from unadjusted p-values (red) and FDR-adjusted p-values (blue). P-value labels represent p-value cutoffs used to estimate the true positive rate and false positive rate for the corresponding region of the Pearson ROC curve.

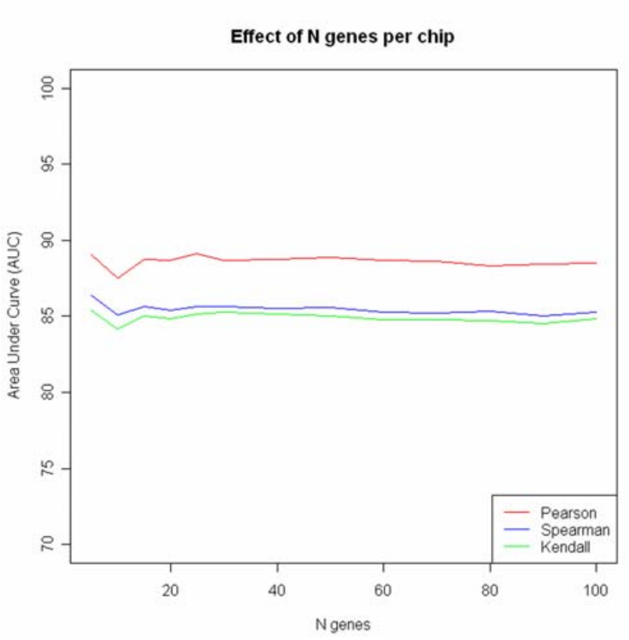

Figure S6. Effect of increasing the number of genes per simulated microarray chip on ROC AUC for DAPfinder with n = 10 chips per class, 250 simulation runs and constant delta = $r_i - r_j$ = +0.5 – (-0.5) = 1.

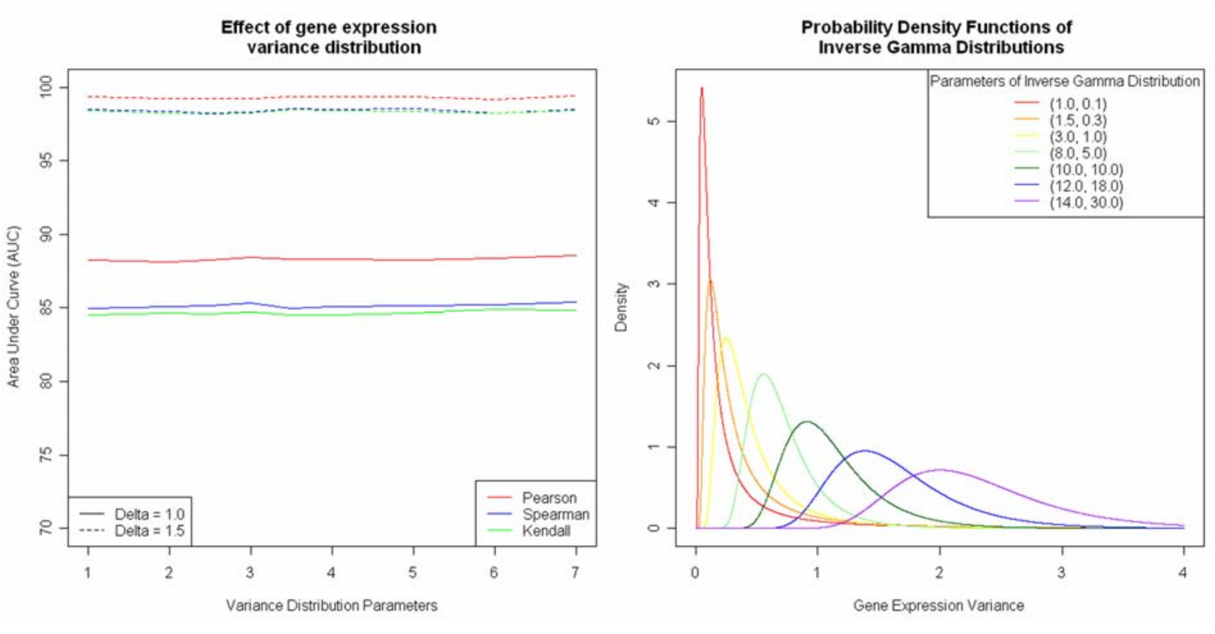

Figure S7. **Left**. Effect of increasing gene expression variance on ROC AUC for DAPfinder with 40 genes per chip, 250 simulation runs, n = 10 chips per class and constant delta = $r_i - r_j$ = +0.5 – (-0.5) = 1. **Right**. Effect of seven Inverse Gamma distribution parameter combinations on distribution of gene expression variances in the simulated microarray data.

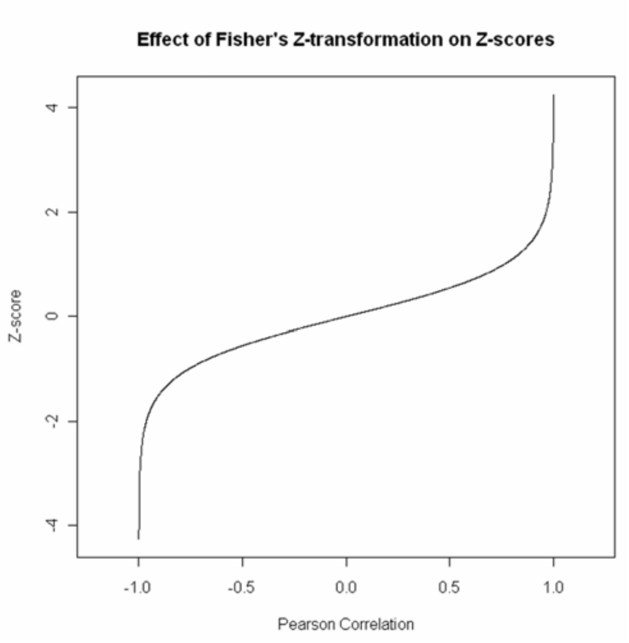

Figure S8. Nonlinear effect of Pearson correlation coefficient strength on Fisher's Z-transformation.

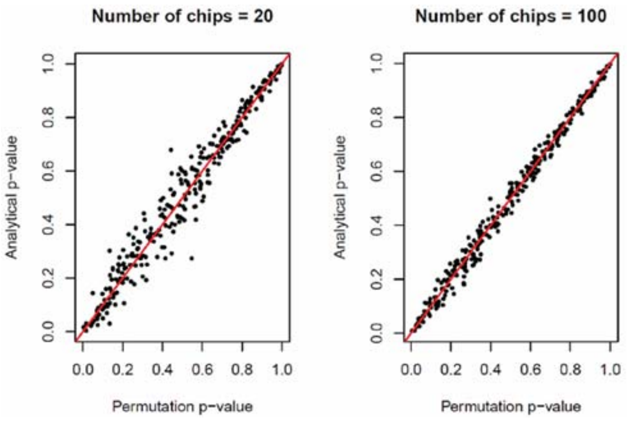

Figure S9. Relationship between analytical p-values (Fisher's Z test) and permutation p-values for sample sizes n = 20 (left) and n = 100 (right).
